# Developing publicly acceptable tree health policy: public perceptions of tree-breeding solutions to ash dieback among interested publics in the UK

**DOI:** 10.1016/j.forpol.2017.03.002

**Published:** 2017-07

**Authors:** Paul R Jepson, Irina Arakelyan

**Affiliations:** School of Geography and the Environment, University of Oxford, South Parks Road, Oxford OX1 3QY, United Kingdom

**Keywords:** Tree health policy, Ash dieback, *Fraxinus excelsior*, Public perceptions, Environmental politics

## Abstract

The UK needs to develop effective policy responses to the spread of tree pathogens and pests. This has been given the political urgency following the media and other commentary associated with the arrival of a disease that causes ‘dieback’ of European Ash (*Fraxinus excelsior*) - a tree species with deep cultural associations. In 2014 the UK government published a plant biosecurity strategy and linked to this invested in research to inform policy. This paper reports the findings of a survey of informed UK publics on the acceptability of various potential strategies to deal with ash dieback, including “no action”. During the summer of 2015, we conducted a face-to-face survey of 1152 respondents attending three major countryside events that attract distinct publics interested in the countryside: landowners & land managers; naturalists and gardeners.

We found that UK publics who are likely to engage discursively and politically (through letter writing, petitions etc.) with the issue of ash dieback a) care about the issue, b) want an active response, c) do not really distinguish between ash trees in forestry or ecological settings, and d) prefer traditional breeding solutions. Further that e) younger people and gardeners are open to GM breeding techniques, but f) the more policy-empowered naturalists are more likely to be anti-GM. We suggest that these findings provide three ‘steers’ for science and policy: 1) policy needs to include an active intervention component involving the breeding of disease-tolerant trees, 2) that the development of disease tolerance using GM-technologies could be part of a tree-breeding policy, and 3) there is a need for an active dialogue with publics to manage expectations on the extent to which science and policy can control tree disease or, put another way, to build acceptability for the prospect that tree diseases may have to run their course.

## Highlights

• A survey at UK countryside events found that publics expect relevant authorities to intervene to manage ash dieback disease.
• Breeding disease tolerance within native ash using traditional methods was the most acceptable solution.
• However, we found substantial levels of support for cis-genetic breeding solutions, but less for trans-GM approaches.
• Support for GM approaches was higher among gardeners and younger respondents.
• Tree health policy in the UK should retain an active intervention component involving development of tree-breeding solutions.

## 1 Introduction

The spread and establishment of tree pathogens and pests beyond their natural ranges has potentially serious consequences for tree health and therefore also for ecology, economy and society (Brasier, 2008). Such incursions are increasing at a time when plants are stressed by factors such as climate change, habitat fragmentation and development (Webber, 2010). This dynamic is leading to growing concerns relating to tree health (Sutherland, 2008, Budde et al., 2016).

Effective and acceptable policy responses to the threats posed by tree diseases are difficult to formulate. This is on account of the reality that: a) dispersal pathways for pest and pathogens are numerous, poorly known and many are beyond human management control (e.g. air borne diseases), b) trees are located (grow) in many different ownership, cultural and policy contexts, c) the issue attracts the interest of multiple policy lobbies due to the multiple identities that trees possess (e.g. as timber resources, components of ecosystems, and symbols of nation, heritage and/or landscape), and d) the limited contribution of silviculture to developed economies means that this area of policy is often under resourced.

UK policy makers faced the arrival and subsequent spread in the UK of *Hymenoscyphus pseudoalbidus* (a fungal disease also known as Ash dieback (ADB) and *Chalara*) that causes high levels of mortality in Ash (*Fraxinus excelsior*). In the UK European Ash is a well-known and loved tree species on account of its cultural, spiritual and literary associations, and its use as a timber and fuelwood source (Rackham, 2014). Confirmation that this disease was the cause of dieback of several hundred ash trees provoked a spike of media reporting between September and December 2012. Media headlines framed the consequences as potentially worse than those of Dutch elm disease, a virulent strain of which killed an estimated 25 million *Ulmus procera* trees across the UK during the 1970s (Forestry Commission, 2016a). Such reporting and associated commentary generated intense pressure on the government to explain the perceived policy failure and ‘do something’.

Tree diseases are nothing new in the UK (Brasier, 2008), however from media and communication perspective (Hansen, 2010), ash dieback and by extension tree health was constructed as a problem of public concern in late 2012. It is likely that the potential impacts of the disease were amplified by a) a recent and successful campaign against the government proposal to ‘sell-off’ publicly owned woodlands in England, and b) the attention and priority given to media reports by government agencies and environmental bodies which legitimised and reinforced the notion of an impending crisis (Potter and Urquhart, 2016;). In short, this case shows that tree health policy is complex and uncertain and can suddenly become political.

In response to rising concern over tree diseases, the UK government established a Tree Health and Biosecurity Expert Task Force. Biosecurity refers to approaches to minimise harm from biological invasions including the spread of pests and diseases (Waage and Mumford, 2008). In 2014 the UK government published its Plant Biosecurity Strategy for Great Britain (Department for Environment, Food and Rural Affairs, DEFRA, 2014). This adopted a risk based approach and included an aim to increase social, environmental and economic resilience to pests (p6) and a specific action to ‘build resilience and learning to live with pests’ (p10). Linked to this strategy, in 2014 the UK Biotechnology and Biological Sciences Research Council (BBSRC), Natural Environment Research Council (NERC), Department for Environment, Food and Rural Affairs (DEFRA), the Forestry Commission, and the Scottish Government invested £7 million in seven research projects across the Living With Environmental Change (LWEC) Tree Health and Plant Biosecurity Initiative (THAPBI) to generate nature and social science knowledge to ‘*inform the development of innovative ways of addressing current and emerging threats to trees and woodland ecosystems from pathogens and pests*” (BBSRC, 2013).

This paper reports the findings from a component of a BBSRC-funded research project that aims to develop new approaches for identifying genes conferring tolerance to ADB and as part of this understand the public acceptability of genetic solutions to tree health issues. Specifically, we report the findings of a survey of public attitudes to different approaches towards developing disease tolerant ash trees, ranging from traditional tree breeding to genetic modification (GM).

In representative democracies achieving congruence between public preferences and policy is of particular importance (Wlezien, 2016). Where policy involves new scientific intervention it is important to investigate public attitudes and preferences in order to identify and understand potential concerns and to build an effective public policy dialogue. The introduction of the agricultural GM technology in the 1990s generated a stark public and political controversy that has generated a persistent negative framing of GM technologies and presents a cautionary tale on how not to introduce a new biotechnology. In their astute analysis of the GM experience, Kearnes et al. (2006) pointed to the need for researchers to bring an understanding of ‘societal imaginations’ relating to their technologies into dialogue with their visions of how their science might solve social and/or policy problems. The present study picks up on this call for ‘upstream steers’ to scientists on the public acceptability of applied science solutions.

This study, as well as contributing to policy development on tree health and the extent to which agricultural GM concerns spill over into silviculture, contributes to a growing academic literature on the design and efficacy of science-policy interfaces (SPI). Briefly, ideas that scientific legitimacy is predicated on neutrality and objectivity gave rise to the belief that science should be separated from politics and ‘speak truth to power’ (e.g. Sutherland et al., 2012). In practice SPIs rarely operate in this linear model and many argue that they should not: that effective SPIs involve dialogue between networks involving scientists and other actors involved in the policy process (see e.g. Koetz et al., 2011, Young et al., 2014). Information on the values and attitudes of citizens typically access the SPI though interest group advocacy, opinion makers and/or commissioned studies. An innovation of this study is to bring public attitudes more closely into the scientific research component of a SPI.

To our knowledge, this is the first attempt to explore the attitudes of the British public towards the development of disease tolerant transgenic trees, and to explore the factors affecting their attitudes. As such, it represents one contribution to future policy guidelines regarding the development and introduction of disease tolerant GM trees.

## 2 The spread and impact of ADB

During the last 20 years populations of European ash (*Fraxinus excelsior*) have suffered damage from the invasive pathogenic fungus *Hymenoscyphus fraxineus*. Although the introduction history is not very clear, the pathogen was most likely introduced from Asia to Eastern Europe through movement of *Fraxinus mandshurica* stock that led to a host shift to *F. excelsior* (Drenkhan et al., 2014, Budde et al., 2016). First reports of the disease came from Poland in 1992, where it has since caused a large-scale decline of ash trees (Hantula et al., 2008), and in the following 2 decades the disease spread across Europe. By the mid 1990s it was also found in Lithuania, Latvia and Estonia (European and Mediterranean Plant Protection Organization, EPPO, 2010). In Denmark, where the disease was first observed in 2002, it had spread to the whole country by 2005 (EPPO, 2010). By 2008 the disease was also discovered elsewhere in Scandinavia, the Czech Republic, Slovenia, Germany, Austria and Switzerland (EPPO, 2010). By 2012 it had spread to Belgium, France, Hungary, Italy, Luxembourg, the Netherlands, Romania, Russia, Britain and Ireland (EPPO, 2010). Ash dieback was first identified in Great Britain in 2012 (Forestry Commission, 2016b).

The disease affects trees in all settings: forest, urban and nursery and causes leaf loss, crown dieback and bark lesions in affected trees (Forestry Commission, 2016b). Infection rates are substantial, particularly amongst young trees: Husson et al. (2012) reported infection in 92% of 2400 trees surveyed across 60 forest plots in France, and in two test plantings of 6000 trees in Denmark, < 5% of trees remained healthy 10 years after planting (McKinney et al., 2014, Budde et al., 2016).

The experience of managing ash dieback in Europe has been negative so far, with most affected countries failing to control its spread, largely due to the absence of effective strategies for managing the disease (European and Mediterranean Plant Protection Organization (EPPO), 2010, Hantula et al., 2008). Even if effective strategies are identified, the process of restoring the ash tree population across Europe with resistant trees is likely to take decades (European and Mediterranean Plant Protection Organization (EPPO), 2010, Hantula et al., 2008).

The first reported incidence of ADB in Britain involved a consignment of infected trees transported from a nursery in the Netherlands to one in Buckinghamshire in February 2012 (Forestry Commission, 2016b). However, confirmation in October 2012 of cases of ADB in established woodland sites in the eastern counties of Norfolk and Suffolk suggest it may have arrived naturally earlier and remained undetected. Since 2012, the disease has spread across Britain: as of 2016 there were 734 confirmed infection sites covering 25.9% of the country, but particularly affecting trees in Eastern and South-Eastern England and Eastern Scotland (Forestry Commission, 2016b). The full environmental, social and economic impacts of ADB in Britain are not yet clear, but based on experience from continental Europe, there is no doubt that the disease has potential to cause significant damage to Britain's ash population (Forestry Commission, 2016b).

An on-line survey conducted in 2014 found high levels of concern relating to tree health in the UK: 73.9% of respondents identified themselves as “concerned” or “very concerned” about the threat of pests and diseases to UK trees and woodlands (Fuller et al., 2016). However, awareness of newly introduced pests and diseases was low. Just, 30.1% of respondents checked that they had heard of ash dieback, and 80.6% checked that either they had heard of the disease but had no knowledge about it or that they had never heard about the disease.

## 3 Tree-breeding solutions and the GM issue

One option for building resilience to ADB in the British landscape is to (re)plant trees with traits that confer low susceptibility to the disease. In conjunction with nationally recognised experts (including experts in phylogenomics of ash tree, plant scientists, and foresters) we identified the following seven approaches for implementing such a policy (which are not mutually exclusive) based on the source and means of production of tree stock:

1. Planting different native (broadleaf) tree species (e.g. oak) to replace ash trees.
2. Planting non-native species of ash trees (e.g. Manchurian ash trees) that are more tolerant to ash dieback.
3. Breeding native tolerant ash trees using traditional techniques.
4. Cross-breeding native ash tree with non-native ash tree to create disease tolerant hybrids.
5. Using accelerated (genomic) breeding to breed native tolerant ash trees.
6. Genetically modifying native ash trees to enhance disease tolerance using cis-genetics (transferring genes between different species of ash trees).
7. Genetically modifying native ash trees to enhance disease trans-genetics (introducing genes from another plant or animal species into ash trees).

An 8th option considered in this study was doing nothing and letting nature take its course, which implies that disease tolerant *F. excelsior* may develop naturally over the next few decades. The assumption is that the natural selection pressure will be very high, selecting for trees that carry existing ‘tolerance’ genes and they will come to dominate *F. excelsior* population over time.

Each of the above options has different characteristics in terms of cost, time to delivery, chance of success, and public acceptability. Silviculture colleagues consulted estimate that options 3 and 4 might take 20–30 years with outcomes uncertain. Screening seedlings for desired genetic markers at an early stage could accelerate the breeding of disease tolerant ash which could be achieved in 10–15 years. Most recently, Harper et al. (2016) identified genetic markers which could be used for development of accelerated breeding. GM approaches offer a more certain and rapid response, probably on a 5–10-year time scale, along with the potential to introduce other desired characteristics such as enhanced carbon sequestration and accelerated growth properties (Farnum et al., 2007, Mathews and Campbell, 2000).

Although genetic modification is now common-place in agricultural crops its application in silviculture is still new despite the potential economic, biosecurity and other advantages GM trees might bring (Institute of Forest Biotechnology, 2007, Nonic et al., 2012, Kazana et al., 2015). Whilst a considerable body of research on GM crops has yet to establish significant food safety and health risks (Kazana et al., 2015) some studies have shown that GM crops may have unintended environmental consequences leading to long-term negative impacts on agro-ecosystems (Prakash et al., 2011, Fernandez-Cornejo et al., 2014). The development of tree-breeding solutions to pest and pathogens using GM techniques is constrained by technical and financial limitations and restrictive regulatory frameworks in many countries, but also socio-economic considerations and in particular the issue of public acceptance (Sedjo, 2004a, Kazana et al., 2015). One of the most significant socio-economic concerns relates to the issue of risk. The introduction of GM trees into the wider environment, whether in woodlands or commercial plantations, also has a potential to be controversial and to face non-acceptance by public (Nonic et al., 2012, Sedjo, 2004b, Tsourgiannis et al., 2015). This represents a risk for science and policy because research investments in technological solutions may not generate policy value. For example, in 2001, in a study funded by the Forestry Commission, scientists at the University of Abertay in Dundee created a batch of GM disease-resistant elm trees (UniSci, 2001). However, due to the lack of general public support to planting out GM elm trees the project has never come to fruition.

Surveying public attitudes to different solutions provides a means to understand this risk and the process of doing so may increase public acceptability of technological solutions through the opportunity to conduct an evidenced-based discussion with the public. Whilst there are surveys of researchers on issues related to research on transgenic trees, there are few surveys of public perceptions of transgenic trees. Most recently, a major survey of the public acceptability of planting transgenic American chestnut (*Castanea dentata*) was conducted by the Oregon State University in the US in 2015 (Needham et al., 2015). The team explored public attitude to the blight resistant chestnut trees developed by the introduction of a single gene from wheat (trans-genic approach). Preliminary findings showed that support for GM isinfluenced by environmental values, perceptions of risk, and demographic characteristics (Needham et al., 2015). However, evidence of public acceptability is only one of the conditions that should be met in order to justify widespread planting of GM trees in the wider environment (Powell, 2016). Additional evidence on public perceptions towards GM trees is limited: a cross-country survey of a number of European and non-European countries found that majority of respondents approved of the use of trans-GM trees in plantations and expressed a willingness to buy trans-GM forest products (Kazana et al., 2015). Similarly, a survey of forestry and applied ecology students in Belgrade found that more than half supported commercial planting of GM trees (Nonić et al., 2015) and that the majority acknowledged the importance of GM in forest research, whilst a study on public attitudes to transgenic forest trees and their products in Greece found that potential negative environmental impacts elicited most concern (Tsourgiannis et al., 2015).

The research reported here adds to this important and emerging area of forest policy research through a case study of a tree species with multiple identities (e.g. cultural, spiritual and material ash, see Rackham, 2014) and uses affected by a new and potentially devastating disease and the context of a culture where GM technology is perceived to be politically controversial. It is intended to provide science and policy a steer on which tree-breeding solutions to Ash dieback, including GM, would be acceptable and for whom.

## 4 Methods

### 4.1 Design and administration of survey instrument

Our aim was to survey attitudes of British publics likely to be interested in the fate of ash trees and who might engage discursively and/or politically with tree health issues. We considered that an effective and resource efficient way to do this would be to conduct a face-to-face questionnaire survey at a number of major countryside events. We took the view that a representative sample of our target population would attend such events. We selected three events to survey that represent the range of engagements with trees, plants, nature and landscape, namely, 1) the Country Landowners Association Game Fair, a three day event that attracts ca. 150,000 visitors with interests in land management and field sports, the majority of whom live or work in the British countryside (surveyed 31 July – 2 August 2015 at Harewood House, Yorkshire), 2) the British Birdwatching Fair, a three day event that attracts ca. 20,000 visitors interested in bird-watching and natural history, and likely to be members of the RSPB and Wildlife Trusts (surveyed on 21–23 August 2016 at Rutland Water, Rutland), and 3) the Royal Horticultural Society (RHS) Wisley Flower show, a six day event that attracts thousands of visitors with interests in gardening and horticulture (surveyed 8–11 September 2015 at the RHS Wisley headquarters, Surrey).

Permission to conduct the survey was obtained from the event organisers and we agreed not to approach visitors when they were engaging with exhibitors or exhibits. We developed a short questionnaire survey that could be completed in 7–10 min whilst people were picnicking, at children's play areas or queuing for exhibits. The questionnaire was developed over a 6-week period with input from project colleagues and developers of other woodland surveys and piloted on 20 people in Oxford parks. It was organised into two sections with a total of 18 questions. The first section contained a question set focused on the knowledge of tree species (ability to identify four common species from photos) and tree diseases, attitudes to ash dieback, respondents' likelihood of accepting different solutions to deal with the disease, and public attitude to genetically modified food and crops. The second section collected information on key socio-demographic elements including age, gender, education level, area of work, source of information on countryside related issues and membership of various countryside related charities and NGOs.

We aimed to survey 400 people at each event based on Dillman et al. (2014) calculation that a sample size of 384 respondents can be projected to a population of ≥ 1,000,000 people with a confidence interval of 95%. A quota sampling strategy was adopted to ensure at least 20 respondents from each age group and a comparable number of men and women in the sample.

The questionnaire was administered by a team of 4 trained enumerators on the dates stated above. Enumerators briefed respondents on what each method entailed and the time frame involved. The majority of respondents were aware of key concepts such as GM, cross-breeding etc.).

### 4.2 Data analysis

Data were analysed using Statistical Package for Social Sciences (SPSS) 22.0 software. Three complimentary analytical approaches were deployed i) basic descriptive statistics, ii) chi-square tests to investigate the relationship between variables and iii) ordinal logistic regression modelling to understand what drives the likelihood of a respondent accepting GM tree-breeding solutions in natural woodlands (Model 1) and forestry plantations (Model 2).

Two models were developed that deployed as the dependent variable the Likert-scale questions on degree of acceptability of GM tree-breeding solutions in the two situations and the same set of 7 explanatory categorical variables (Table 7). These included six variables that showed significant chi-square association with the dependent variables, along with GENDER (7th explanatory variable), which despite not showing a significant chi-square association with the independent variables, was included based on the hypothesis that it might interact with the six other variables to influence model outcomes.

For the purposes of data analysis the explanatory variables MEMBERSHIP and AGE were recoded. The eleven membership organisations were grouped into four broad categories based on practises of engaging with nature and landscape: land-management, wildlife and natural history, heritage, and gardening. Age-groups were combined into generational categories, namely pre-war generation (born before 1945); baby boomers (born between 1945 and 1965); generation X (born between 1965 and 1982) and millennials (born between 1982 and 2000). Summary hypotheses for the selected variables are presented in Table 1.

**Table 1:** Summary hypotheses (independent variables) used in two ordinal logistic regression models developed to explain drivers of attitudes to GM tree-breeding solutions in different settings.

Variable	Hypothesis
X_1_: GENDER	Gender does not play a significant role in attitude to tree breeding solutions.
X_2_: GENERATION	Respondents in older generation categories are more likely to be against GM solutions to ash dieback
X_3_: EDUC	Respondents with a degree are more likely to be pro-GM
X_4_: VIEW	Respondents who are concerned or very concerned about ADB are more likely to be in favour of GM solutions to ADB
X_5_: GMFOOD	Respondents who support GM-food and crops are more likely to be in favour of GM solutions to ADB
X_6_: PUBLICSAY	Respondents who think their opinion should be taken into account in decision making, are less likely to support introduction of GM trees
X_7_: EVENT	Respondents who attended the first two events (the Game fair and the Bird fair) are more likely to be anti-GM solutions whilst those attending the RHS Wisley Flower Show are more likely to be pro-GM

## 5 Results

### 5.1 Respondent profile

We achieved Dilman's 384 respondent target at each event and 1152 respondents were surveyed in total (https://doi.org/10.5287/bodleian:7egjVbP0J). The visitor demographic of the events was clearly skewed towards older (46% > age 61) and retired (44.6%) but the overall gender and age group quotas were obtained. Our overall sample at the Bird Fair and Wisley Flower Show contained more retired people (47.2% and 62.2% of respondents respectively) compared with the CLA Game Fair (20.6%). Based on non-retired respondents the CLA Game fair was attended by a significant number of people working in the manufacturing, construction, agriculture and forestry sector (40.5%, *n* = 247) compared with the Bird fair (16.9%, *n* = 195) and the Flower show (17.3%, *n* = 139). Respondents were significantly better educated than the general UK population: 49.6% held a university degree or higher compared with 27.2% of general population in England and Wales (Office for National Statistics, ONS, 2014). There was no significant difference in the proportion of respondents with a university degree-level across the three events.

The overwhelming majority of respondents were members of at least 1 countryside organisation (85.9%) and 52.7% of all respondents were members of 2 or more (Table 2). Our findings on membership aggregated by four broad organisational types confirm our prediction (Methods) that each event has a distinct membership profile: CLA Game fair attendants had a high likelihood of being members of land-based charities and associations, Bird fair attendants - of wildlife and natural history related associations, and Flower show attendants - of gardening charities and associations (*p* < 0.001 for all three events). Thus event participation can be taken as a proxy for a particular type of interested public.

**Table 2:** Membership of categories of UK countryside and nature charities.

Category organisation	Game fair	Bird fair	Flower show
Land-based (CLA, Game Conservancy Trust, NFU) (*n* = 173)	94.8% (164)	1.7% (3)	3.5% (6)
Wildlife and natural history (RSPB, Wildlife Trust, Plantlife) (*n* = 651)	7.5% (49)	75.0% (488)	17.5% (114)
Landscape and heritage (National Trust, Woodland Trust, English Heritage, CPRE) (*n* = 712)	20.9% (149)	37.1% (264)	42.0% (299)
Gardening (RHS) (*n* = 334)	10.8% (36)	12.0% (40)	77.2% (258)

TV programmes and newspapers were the most important source of information on countryside issues for most respondents, but a significant proportion of respondents obtained information from the other categories including scientific and government reports (Fig. 1, Supplementary material). We found no significant difference between the respondents at the three events with regards to their preferred source of information on countryside issues.

### 5.2 Knowledge of trees and diseases

Tree identification knowledge measured by the ability to correctly identify the ash tree (*F. excelsior*) from 4 tree photo-choice options was high across the three events (mean 64.7%) and highest amongst Bird Fair publics (77.5%, Table 3), and, correspondingly, highest amongst members of Wildlife and Natural history associations (> 80%).

**Table 3:** Respondents' response to a question set measuring knowledge of tree identification and awareness of tree diseases effecting *F. excelsior*, according to event.

	Game fair (*n* = 384)	Bird fair (*n* = 384)	Flower show (*n* = 384)	*χ*^2^
Correct identification of ash tree photo	54.5%	77.5%	62.1%	58.471⁎⁎⁎
Heard about ash dieback	85.2%	95.8%	89.8%	25.052⁎⁎⁎
Heard about *Chalara fraxinea*	12.8%	25.3%	13.3%	26.989⁎⁎⁎
Heard about emerald ash borer	19.3%	26.0%	14.9%	15.114⁎⁎

⁎⁎ *p* < 0.01.

⁎⁎⁎ *p* < 0.001.

The overall knowledge of trees and ash dieback was high, but varied according to event, age, and professional occupation (Table 1, Supplementary material). There was a significant difference between age groups, with older respondents being more likely to correctly identify the ash tree (*p* < 0.005). Amongst the employed publics, respondents employed in the public and education sectors had the highest level of knowledge – 66.4% of respondents from this sector were able to correctly identify the ash tree, closely followed by 64.5% of respondents from the manufacturing, construction, agriculture and forestry sector. The level of knowledge was highest amongst the retirees - 69.5% of retired people correctly identified the ash tree. These figures are significantly higher than similar figures for the general UK population. A comparable question in a UK wide survey conducted by Woodland Trust found 17% of all respondents, and just 10% of 18–24 year olds could identify an ash leaf (YouGov/Woodland Trust, 2013).

In our study, a high proportion of respondents had heard about either ash dieback (mean 90.3%) or *Chalara* (mean 17.1%), with a significant difference between events (Table 3). Again, the respondents at the Bird Fair had the higher level of knowledge of ash dieback and *Chalara* compared to respondents from the other two events - 95.8% and 25.3% respectively. Knowledge of the Emerald Ash borer amongst all respondents was significantly lower than that of ash dieback (20.1% across the three events). Chi-square tests found significant association between knowledge of ash trees and associated diseases, with the Bird Fair public being the most knowledgeable and CLA Game fair public being the least knowledgeable. Moreover, we found a significant correlation (*p* < 0.001) between age group, occupation and awareness of ash dieback: amongst the age groups, 30–40 years olds were the least knowledgeable and in terms of work area the individuals employed in the health, forestry/agricultural sectors, as well as retired people were most knowledgeable (Table 1, Supplementary material). It should be noted that when compared to the general population our event publics were highly knowledgeable. In a survey of 2000 respondents sampled from the general population Fuller et al. (2016) reported low levels of knowledge of a number of tree diseases including ash dieback, with 69.9% of all respondents reporting that they either had “heard of but had no knowledge” or had “never heard of the disease”.

### 5.3 Attitudes to solutions to ash dieback

Across the three events the majority of respondents (82.9%, *n* = 953) answered that they would be concerned if ash trees disappeared from the British countryside, with older respondents more likely to be concerned (*p* < 0.001) (Table 4). Further, the results showed a significant association between the type of event, and attitude to ash trees, with the Bird Fair recording the highest proportion (89.3%) of respondents who said they would be concerned or very concerned if ash trees disappeared from the British countryside. Further, respondents in all occupational groups and with a membership of any organisation reported almost equally high levels of concern.

**Table 4:** Attitude to ash trees, by age and type of event (numbers of respondents in brackets).

Answer options	If ash trees disappeared from the British countryside I would not be concerned	I would not be concerned if replaced by other species	I would be (very) concerned if ash trees disappeared from the British countryside	I don't have an opinion about this
Respondent's age				
Under 21	0.0% (0)	18.2% (4)	68.2% (15)	13.6% (3)
21–30	2.2% (2)	8.7% (8)	81.5% (75)	7.6% (7)
31–40	5.1% (4)	16.5% (13)	63.3% (50)	15.2% (12)
41–50	1.3% (2)	6.4% (10)	82.2% (129)	10.2% (16)
51–60	5.3% (14)	7.5% (20)	85.3% (227)	1.9% (5)
61–70	2.2% (8)	8.8% (32)	84.8% (308)	4.1% (15)
71–80	2.3% (3)	8.4% (11)	86.3% (113)	3.1% (4)
> 81	0.0% (0)	10.3% (3)	89.7% (26)	0.0% (0)
Event				
Game fair	2.6% (10)	8.9% (34)	81.0% (311)	7.6% (29)
Bird fair	2.9% (11)	4.7% (18)	89.3% (342)	3.1% (12)
Flower show	3.1% (12)	12.8% (49)	78.3% (300)	5.7% (22)

In 2 separate questions, respondents were asked to rank their 3 most preferred (top) and 2 least preferred (bottom) solutions to ash dieback: the first question (Q.5 in the questionnaire) contained a list of 8 different options, the second question (Q.6) contained the same options, but with the addition of the potential time scale needed to develop each option. In both cases, the *do nothing* option received little support. It appeared as one of the two least preferred options (from a list of 8) of 82.9% of all respondents and the 1st, 2nd or 3rd choice for < 10% of respondents (Table 5). Breeding native ash either through conventional means or accelerated breeding were by far the most preferred options: these two choices appeared in the top three of 95.1% and 96.6% of all respondents respectively. Prior to learning of the time associated with each option, breeding a native ash using conventional means was the first choice option of 68.8% (720) and accelerated breeding of native ash the second choice of 55.5% (487) (Table 2, Supplementary material). When respondents were informed of the time scale (25 + years, 15 years respectively), the proportion of respondents who selected natural breeding dropped significantly to 48.3% (422 respondents) and accelerated breeding to 42.2% (*N* = 360). However, both remained the most preferred first and second tree-breeding options.

**Table 5:** Preferred solutions to ADB after respondents informed of approximate timescale of actions (*the figures in bold show the options which were selected by the highest proportion of respondents for each category*) (number of respondents reported in brackets).^a^

Course of action	1st choice	2nd choice	3d choice	2nd least preferred choice	Least preferred choice
No action (let nature take its course)	7.3% (63)	2.3% (20)	7.5% (64)	28.3% (243)	**54.6% (469)**
Plant a different tree species, e.g. oak	17.7% (123)	19.5% (135)	22.2% (154)	27.6% (191)	13.0% (90)
Plant different non-native species of ash	19.4% (99)	21.7% (111)	22.3% (114)	22.7% (116)	13.9% (71)
Breed native tolerant ash	**48.3% (442)**	27.5% (252)	19.3% (177)	3.3% (30)	1.5% (14)
Accelerated breeding of native tolerant ash	34.2% (292)	**42.2% (360)**	20.2% (172)	2.3% (20)	1.1% (9)
Cross breed native ash with non-native ash	4.8% (23)	23.6% (113)	**50.8% (243)**	14.2% (68)	6.3% (30)
Cis-genetics	13.9% (84)	14.9% (90)	24.9% (151)	37.6% (228)	8.7% (53)
Trans-genetics	1.4% (9)	6.9% (44)	7.2% (46)	**55.2% (353)**	29.4% (188)

a The proportion of respondents who selected various options as their 1st, 2nd and 3d choice does not necessarily have a decreasing order; for instance, there are more respondents who ranked cross-breeding as their 3d choice than respondents who ranked accelerated breeding as their second choice. However, the majority of respondents ranked accelerated breeding as their second choice hence this option takes a priority. The same applies to the ranking of the two least preferred options.

The third and fourth most popular options, as a first choice, were planting non-native ash trees to replace diseased native ash (19.4%), and planting different native trees species to replace ash (17.7%). These two options are management options, rather than tree-breeding options; however, this choice shows that informed publics are not generally negative about a change in trees species which could result in a different composition of British forests within the next few decades.

Creating a disease tolerant ash tree using trans-genetics was the least preferred tree-breeding option for 55.2% of respondents (*n* = 353) making it as one of their two least preferred options for the majority of respondents. It did appear as one of the top 3 options, but was supported only by 15.5% of all respondents. However, the cis-genetics option was chosen as one of the three preferred options by 34.1% of respondents (Q.5) which rose to 53.9% when informed of the time scale (< 10 years) for achieving an outcome (Q.6), with the proportion of people ranking it as the first choice increasing from 3.9% to 13.9%.

The option to cross-breed native ash with a non-native ash tree was ranked as a third preference by 56.5% (*N* = 315) of all respondents and this proportion dropped only slightly - to 50.8% (*N* = 243) when informed of the timescale (20 + years). Planting tolerant non-native ash was selected by 63.4% of all respondents as one of their most preferred 3 options; this option was not, however, the most preferred option in any single category.

Overall, we found that learning the time scale did not affect the ranking of top 3 and bottom 2 options but it did change the proportions for each. Table 5 reports the full results when people were informed of time scales (also see Table 2 in Supplementary Material).

The findings showed a significant association between respondents' attributes and attitudes to ash trees, and their top-3 solutions. Those who were more concerned about the ash tree disappearing were more likely to be against the “*no action*” option (*p* < 0.001), younger respondents were more likely to be in favour of methods involving a higher degree of scientific intervention such as GM options, and older respondents were more likely to be in favour of traditional breeding techniques (*p* < 0.001 for both). Furthermore, the Millennials and Generation X respondents were likely to be more in favour of GM approaches, in comparison with the older generations. In addition, respondents with a degree-level education were more likely to give a low preference to the “No action” and two GM options (*p* < 0.001 for all) (Table 3, Supplementary material). Further, in terms of event distribution, respondents who attended Wisley flower show were less likely to be against the GM options and the “no action” option than respondents interviewed at the other 2 events (Table 6).

**Table 6:** Respondents' choice of the most preferred and least preferred options, by type of event.

Name of the event	Potential options to deal with ash dieback				
	No action (as the least preferred option)	Natural breeding (as the most preferred option)	Accelerated breeding (as the 2nd most preferred option)	Cross-breeding (as the 3d most preferred option)	Trans-genetics (as the second least preferred option)
The Game fair	59.4%	47.8%	36.1%	43.4%	56.7%
The Bird fair	52.9%	50.8%	43.1%	53.1%	60.1%
The Flower show	51.4%	46.1%	47.0%	56.6%	48.6%
*p*-value	18.014⁎	9.294	17.790⁎	23.651⁎⁎	24.051⁎⁎

⁎ p < 0.05.

⁎⁎ p < 0.01.

In the final question in Section 1 of the questionnaire (Q.9), respondents were asked whether public should have a say on which tree modification solution is adopted when dealing with the disease (ash dieback. 58.2% (*N* = 667) of respondents thought their views should be taken into account, while 41.8% (*N* = 478) said that decision making should be left to experts.

### 5.4 Attitudes to GM trees

When asked whether they would be willing to accept genetically modified ash trees planted in natural woodlands, or forestry plantations, 37.8% (*n* = 430) of all respondents said they would approve or strongly approve of GM ash trees being planted in natural woodlands (Figs. 2a,b, Supplementary material). This proportion increased significantly to 59.9% (*n* = 680) for the GM ash trees planted in forestry plantations. The majority of respondents who would approve or strongly approve GM ash trees planted either in natural woodlands, or in forestry plantations, were also more likely to give a higher ranking to both cis-genetics and trans-genetics options (*p* < 0.001 for all cases) (Tables 4 and 5, Supplementary Material).

Respondents were further asked their views on GM food and crops, to see whether this has an impact on how they perceive GM trees. 30.6% of all respondents reported being in favour of GM food and crops; 41.7% said they were against, and 27.7% were not sure. Chi-square test results showed a significant positive correlation (< 0.000) between respondents' attitude to GM food/crops and their attitude to GM trees, with those in favour of GM food and crops being more likely to support GM trees both in forestry plantations and in natural woodlands. We further found that respondents' attitude to GM food/crops was significantly correlated (*p* < 0.001) with their ranking of tree breeding solutions, with those in favour of GM food and crops being more likely to give a higher rank to the GM options to deal with ash dieback.

#### 5.4.1 Results of the ordinal logistic regression models

Cumulative odds ordinal logistic regression models were run to determine the effect of a number of independent variables i.e. respondents' gender, generational category, level of education, the type of event attended, respondents' views on ash dieback, attitude to GM food/crops and opinion on public involvement in decision making) on the level of acceptability of planting GM ash trees in natural woodlands (Model 1), and forestry plantations (Model 2, Table 7). For both models, there were proportional odds, as assessed by a full likelihood ratio test comparing the fitted models to models with varying location parameters, *χ*^2^ = 18.509, *p* = 0.185 for Model 1 and *χ*^2^ = 28.610, *p* = 0.347 for Model 2. The deviance goodness-of-fit test indicated that both models were a good fit to the observed data, *χ*^2^ (1818) = 1786.894, *p* = 0.983 for Model 1, and *χ*^2^ (1818) = 1580.750, *p* = 0.869 for Model 2. Further, both final models were statistically significant when tested against the intercept-only model, *χ*^2^ (18) = 253.931, *p* < 0.0005 for Model 1, and *χ*^2^ (18) = 184.307, *p* < 0.0005 for Model 2. In short, both models were found to be robust in explaining what drives attitudes to GM tree breeding solutions in different settings amongst the sample population.

**Table 7:** Results of the two ordinal regression models of the level of acceptability of planting GM ash trees in natural woodlands (Model 1), and forestry plantations (Model 2), based on a number of independent variables.

	Model 1 (introduction of GM trees in natural woodlands)						Model 2 (introduction of GM trees in forestry plantations)					
	Hypothesis test			Exp.(B)	95% Wald confidence interval for Exp.(B)		Hypothesis test			Exp.(B)	95% Wald Confidence Interval for Exp.(B)	
Parameter	Wald chi-square	df	Sig.		Lower	Upper	Wald chi-square	df	Sig.		Lower	Upper
[Event (Game fair) = 1]	13.323	1	0.000	(−)1.296	0.981	1.714	4.526	1	0.001	(−)1.363	1.025	1.813
[Event (Bird fair) = 2]	14.426	1	0.000	(−)1.335	1.020	1.748	4.769	1	0.001	(−)1.358	1.032	1.786
[Event (Flower show) = 3]	.	.	.	1	.	.	.	.	.	.	.	1
[Views = 1 (concerned or very concerned]	12.156	1	0.000	1.543	0.865	2.754	7.227	1	0.001	1.825	0.374	0.967
[Views = 2 (not concerned if replaced by other species)]	13.173	1	0.000	1.538	0.958	2.469	7.778	1	0.001	1.767	0.425	0.850
[Views = 3 (not concerned)]	.	.	.	1	.	.	.	.	.	.	.	1
[Publicsay = 0 (views should be taken into account)]	10.010	1	0.000	(−)1.204	0.074	1.679	12.298	1	0.000	(−)1.369	1.010	1.769
[Publicsay = 1 (leave decision making to experts)]	.	.	.	1	.	.	.	.	.	.	.	1
[Gender (female) = 0]	0.995	1	0.319	1.118	0.898	1.393	0.024	1	0.877	0.983	0.785	1.229
[Gender (male) = 1]	.	.	.	1	.	.	.	.	.	.	.	1
[Education (GCSE) = 1]	6.783	1	0.009	1.700	1.140	2.534	0.029	1	0.864	1.036	0.689	1.559
[Education (vocational training) = 2]	5.324	1	0.021	1.737	1.087	2.776	1.300	1	0.254	1.038	0.819	1.326
[Education (degree) = 3]	15.183	1	0.000	1.557	1.064	2.279	10.035	1	0.022	1.320	0.703	1.533
[Education = 4]	.	.	.	1	.	.	.	.	.	.	.	1
[Generation Y = 1]	13.733	1	0.000	2.727	1.993	3.516	11.233	1	0.001	1.334	0.699	1.808
[Generation X = 2]	25.023	1	0.000	2.205	1.841	2.739	12.012	1	0.001	1.215	0.896	1.987
[Baby-boomers = 3]	22.559	1	0.000	1.581	1.091	2.055	15.338	1	0.000	1.124	0.873	1.692
[Pre-war generation = 4]	.	.	.	1	.	.	.	.	.	.	.	1
[GMfood (support) = 1]	11.829	1	0.000	11.766	11.330	12.168	12.517	1	0.000	3.848	1.098	5.1341
[GMfood (not sure) = 2]	11.184	1	0.000	2.186	1. 611	2.621	10.020	1	0.000	1.305	1.033	1.573
[GMfood (anti) = 3]	.	.	.	1	.	.	.	.	.	.	.	1

For both models, test of model effects showed that the type of event attended, views on ash dieback, public say on decision making, level of education, respondent's generational category and attitude to GM food/crops all had a statistically significant effect on the attitude to GM ash trees. The only independent variable that did not have an effect on the outcome of the dependent variable in both models was gender. For both models, respondents attending the Game fair and the Bird fair had similar - 1.3 higher odds of objecting to the introduction of GM ash trees in natural woodlands and forestry plantations compared to those attending the Flower show.

Respondents who were not concerned about ash dieback provided ash trees were replaced by other species, were 1.5 times more likely to accept GM ash trees being planted in natural woodlands than respondents who were generally not concerned about ash dieback. Respondents who were concerned or very concerned about ash dieback, were also 1.5 times more likely to be in favour of GM ash trees being planted in natural woodlands than those not concerned about the disease (Model 1). In Model 2, respondents who were not concerned about ash dieback provided ash trees were replaced by other species, were 1.7 times more likely to favour the introduction of GM ash trees in forestry plantations than respondents who were generally not concerned about ash dieback, and respondents who were concerned or very concerned about ash dieback, were also 1.8 times more likely to be in favour of GM ash trees being planted in forestry plantations than those not concerned about the disease.

Respondents who thought that their views should be taken into account were 1.2 times less likely to be in favour of GM trees in natural woodlands and 1.3 times less likely to be in favour of GM trees in forestry plantations, than respondents who thought that decision-making should be left to experts. Respondents with a university degree had 1.5 times higher odds of supporting the introduction of GM ash trees in woodlands and 1.3 times higher odds of supporting the introduction of GM ash trees in plantations than people without a degree.

An increase in age (expressed as difference in generations) was associated with a decrease in the odds of the level of acceptance of both the GM trees in natural woodlands and GM trees in forestry plantations. Baby-boomers (51–70 year olds) were 1.5 times more likely to support the introduction of GM ash trees in natural woodlands technologies and 1.1 times more likely to support the introduction of GM ash trees in plantations, than pre-baby boomer generation (71 +) (Model 1). Generation X (31–50) had 2.2 times and 1.2 higher odds and Generation Y (millennials) (under 31) had 2.7 times and 1.3 times higher odds for supporting GM ash trees in natural woodlands and forestry plantations respectively, than pre-baby boomer generation. Overall, the highest support towards GM ash trees for both models was amongst the millennials, and the lowest was amongst the pre-war generation, showing a significant generational gap in terms of attitude to GM technologies.

Respondents who did not have a defined attitude to GM food and crops, were 2 times and 1.3 times more likely to be in favour of the introduction of GM ash trees in natural woodlands and forestry plantations respectively, than respondents who were anti GM food and crops. Respondents who supported GM food and crops, were 11 times and 3.8 times more likely to be in favour of GM ash trees in natural woodlands and forestry plantations respectively, than those who were anti GM food and crops.

## 6 Discussion

Attendees at the events where we surveyed are not a homogenous publics, they come from different walks of life and engage with ash trees and tree diseases in different ways: as land-owners and woodland managers, naturalists and gardeners. However, what unified them in our study was very high levels of concern regarding ADB and strong rejection of the ‘do nothing and let nature follow its course’ option. A clear steer for policy is that this well-educated sub-set of British publics with the interest and time to attend countryside events, and the majority of whom are members of charities/NGOs with policy presence and a strong lobby, have an expectation that the relevant authorities will intervene to manage the impacts of the disease.

The significantly higher proportion of people who could correctly identify ash in our survey is not surprising considering the fact that the respondents were attending countryside events and hence were expected to have a higher level of knowledge regarding the issues related to countryside, in comparison with the general population. A range of tree-breeding solutions were acceptable to the ‘interested’ publics, but breeding disease tolerance within native ash using traditional methods was the most acceptable option amongst these well-educated publics.

The findings also revealed a high level of concern about ash dieback amongst all age groups, which is clear from the fact that the “no action” option was the least popular choice. This shows that the respondents think that it is necessary to act to save native ash trees from dieback. These findings agree with the results of two “Public Opinion of Forestry” surveys conducted by Forestry Commission in 2015 and 2013 in which around three quarters of the representative population of 2000 adults agreed or strongly agreed that ‘*action should be taken by authorities and woodland managers to protect trees from damaging pests and diseases*’ (Forestry Commission, 2015, Forestry Commission, 2013). The results also agree with the findings presented in Fuller et al. (2016) where more than three-quarters of respondents believed that pests and diseases should be managed if affecting the health of native trees.

Results further showed that the proportion of respondents who selected cis-genetics as one of their top three options increased quite substantially - by 19.8% - when respondents were informed of the relatively short timescale associated with this option (5–10 years), in comparison with other options, with the proportion of respondents ranking cis-genetics as their top choice increasing from 3.9% to 13.9%. However, for trans-genetics, this proportion only increased by 5% across the top three options, and only by 0.6% for respondents who selected trans-genetics as the first choice. This shows that overall the respondents were concerned enough about ash dieback to select cis-genetics, i.e. a GM approach, as one of their preferred options to deal with ADB. On the other hand, even the high level of concern about ash dieback was not enough to overcome people's reluctance to choose a more “interventionist” trans-genetics approach, even when informed of the associated timescales. This is likely due to the high degree of scientific intervention which this option entails, which suggests that the respondents prefer to wait for more than a decade longer for the solution to be found as long as it is perceived as a more natural solution.

From a forward-looking policy perspective two findings concerning the level of acceptability of GM techniques (and in particular cis-genetics) amongst Millenials and gardeners are significant, namely: 1) respondents are less averse to GM solutions when asked directly about these compared to when asked to score them in a list of other options including natural breeding; 2) those who are less concerned about ‘nativeness’ (for whom planting disease tolerant non-native ash is acceptable) are more likely to find GM ash trees acceptable as did a substantial proportion of Millennials and gardeners (81.1% of Millennials and 53.4% of gardeners selected cis-genetics as one of their top 3 choices). These findings suggest that going forward developing and planting GM trees may become a publically acceptable solution to deal with tree diseases. This said, our results also showed that members of large natural history NGOs with professional and influential policy teams (such as the RSPB and Wildlife Trusts) were more opposed to GM solution.

### 6.1 Discussion of the model

Overall, the findings for both models are similar, and indicate that the older generation is generally less in favour of GM technologies used for tree breeding, whether in natural woodlands, or in forestry plantations. This might be due to older people being more technology averse, with younger generations, especially the Millennials, growing up in the age of the internet, and being more accepting of technological solutions to natural problems. As could be expected, the findings also show that the higher level of education was associated with a higher level of acceptance of GM solutions to tree breeding, which could be attributed to respondents with degrees being more informed about the benefits of GM technologies, and more in favour of a “quicker” solution to deal with ash dieback. Again, predictably, respondents who expressed concern about ash dieback and those who were more supportive of GM food and crops, were more likely to accept GM solutions, both in natural woodlands and in forestry plantations.

An interesting finding for both models is that the respondents attending both The Game fair and the Bird fair, were generally less inclined to accept GM solutions to breeding tolerant ash trees, than those attending the Flower show. This could be explained by the fact that modification of plants and crops has been used in gardening for centuries, which means that the gardeners in general are more used to the idea of human intervention when growing plants than other respondents.

### 6.2 Limitations of the study

Like any public attitude survey, this survey had its limitations. In particular, it was impossible to capture the complex dimension of the issues within a short-survey delivered while people were on a day out enjoying themselves. The acceptability of tree-breeding solution might change if information is more fully conveyed on the likelihood of success of each solution, the timescale involved and what would be involved in adopting the solution in practice. For instance, one of the proposed options to deal with ash dieback includes replacing diseased ash trees with other native trees; however, given that around 47% of UK woodlands, most in private sector ownership, are currently unmanaged and many lack access (DEFRA, 2013), an effective replanting programme might be hugely costly. Such information, were it available in consolidate form, might increase the public acceptability of the *no action* option. However, we believe that the amount of information provided in our survey was deeper and more systematic than is likely to be communicated via various media and within the attention span of non-specialists. Further, the majority of those interviewed had a keen interest in countryside and already had prior knowledge and understanding of the scientific concepts used in the survey. As such our results offer a robust guide to the likely acceptability of tree-breeding solutions to ash dieback.

## 7 Conclusions

Developing and planting out disease resistant varieties of ntative trees is one means to build resilience and learning to live with tree pests and pathogens. The aim of this study was to provide tree health policy and science a ‘steer’ by surveying attitudes of interested publics to a range of tree-breeding solutions to ADB. We posited that publics attending countryside events will be more invested in this area of policy in terms of their level of knowledge and concern, and more able to express a policy preference by virtue of their potential engagement with NGOs and campaign groups. This was borne out by our results: the majority of respondents had heard of ADB and were members of two or more countryside-related NGOs.

Breeding native ash was the most acceptable solution to deal with ash dieback, with other natural options such as accelerated breeding and cross-breeding also gaining high levels of support. However, all tree breeding solutions including GM (in particular cis-GM) had certain levels of public acceptability. Awareness and concern about ash dieback is high amongst the publics. This survey demonstrates an expectation that government will intervene to address the issue.

While fully recognising the complexity of tree health policy, the insights on pubic acceptability of tree breeding solutions reported here suggest that policy will benefit from: 1) retaining an active intervention component involving the development of tree-breeding solutions, and 2) considering management options alongside tree-breeding options, such as large scale replanting diseased ash trees with other native tree species, or non-native ash. Replanting with native and non-native species has significant levels of public acceptability that may increase in the future and offers a more rapid response to the spread of ash dieback.

We also recognise that many experts in this area of policy believe that re-planting with disease-tolerant Ash is unfeasible except in local situations and as a result letting nature taking its course might be the best course of this action (Jepson and Arakelyan, 2016). Given this potential miss-match between such expert policy advice and the public acceptability of the resulting policy (*no action*) option we suggest that interested publics need to be actively engaged in dialogue on tree health policy. Further, we suggest that gardeners would be a good group to engage in such a dialogue, at least initially, given their acceptability of a range of tree-breeding solutions and prominent role of gardening in the UK culture, media and commerce.

Lastly, it is not known to what extent UK publics make a distinction between the use of GM in agri-food production and its use in silviculture. The extensive literature on public perceptions to GM in agri-food production shows that European have more negative views to GM than do North Americans. However, such perceptions are generally associated with food consumption concerns arising from negative press converge and lower trust in regulatory institutions (Frewer et al., 2013) that do not apply to trees. Future research might usefully investigate the relationship between attitudes to GM in agriculture and in silviculture and, for instance the importance of value concerns such as ‘playing God’ and ‘unnaturalness’ in determining public acceptability of deploying GM techniques in tree health policy.

This study acts as a gateway to further research, which will provide insights into the views of other major stakeholders (industry and forestry professionals, media and the government), as well as views of the wider UK population.

## Figures and Tables

**Figure:**
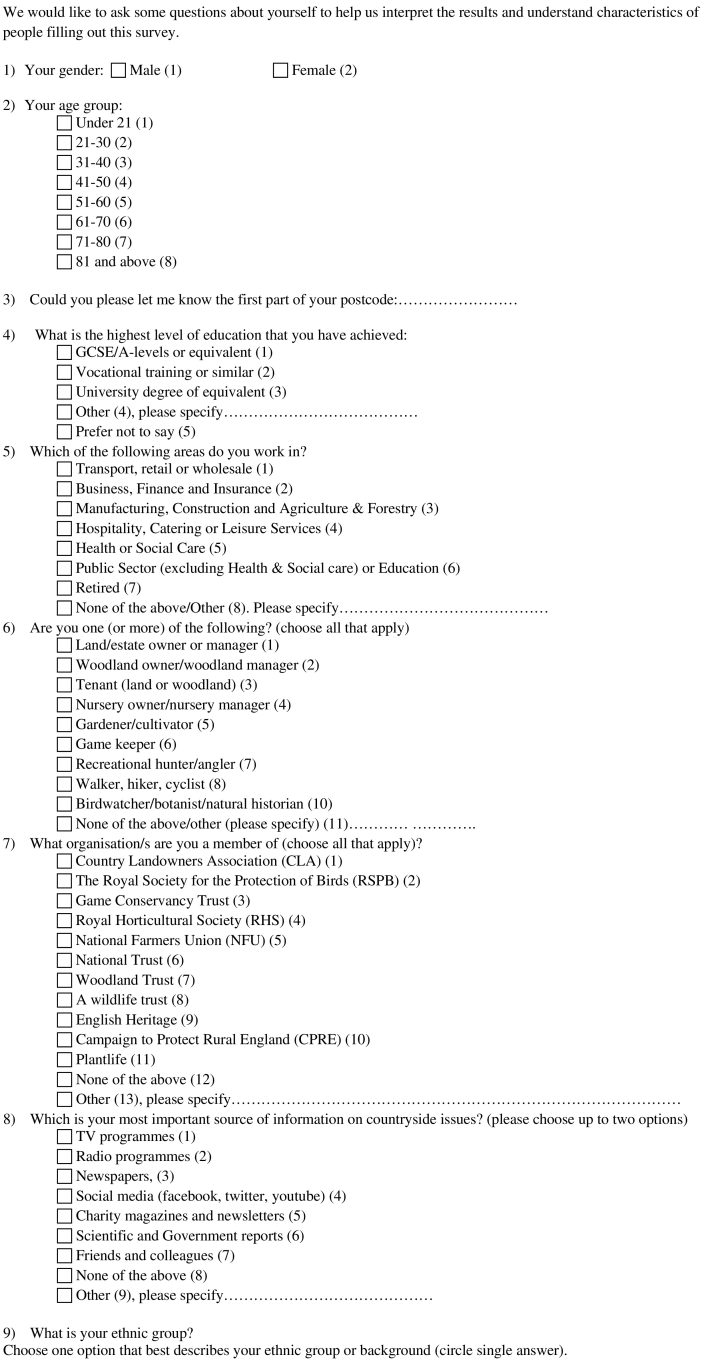


## Appendix 1 Questionnaire on tree breeding solutions

### Section I

1. **Let's start with a quiz. Which of these is the Ash tree?** (4 leaf close-ups of oak, birch, poplar and ash are shown)
2. **To what extent do you agree or disagree with each of the following statements?**
  a. Our woodlands are fragile, and human interference can cause unexpected disaster.
  b. If tree diseases continue on their present course, they will have a catastrophic effect on our landscapes.
  c. Tackling the spread of tree diseases will only be possible if people agree to replace native trees with other disease resistant tree species.
  d. Our trees and woodlands are quite adaptable and will recover from any damage caused by disease.
  e. We do not need worry about tree diseases because science and technology will be able to treat them.
  f. It is possible to avoid catastrophic effects on our landscapes if tree disease problems are managed by experts and scientists.
  g. Tree diseases should be controlled by the government passing laws that control the import and spread of trees and seedlings that might transmit disease.
  h. Landowners should be free to change woodlands in any way they like.
3. **Have you heard of any of these tree diseases?**
  a. Ash dieback Yes/No.
  b. *Chalara fraxinea* Yes/No
  c. Emerald ash borer Yes/No
  d. Do you know any other tree diseases, whether past or present? Please specify…………………………
4. **Which one of the following statements most closely represents your view?**
  a. If ash trees disappear from the British countryside I would not be concerned.
  b. I would not be concerned if ash trees disappear from the British countryside so long as they were replaced by other tree species.
  c. I would be (very) concerned if ash trees disappeared from the British countryside.
  d. I don't have an opinion about this.
5. **What would be your preferred course of action to deal with Ash dieback?**
  a. No action - let nature take its course
  b. Plant a different tree species e.g. oak
  c. Plant different non-native species of ash (e.g. Manchurian tolerant ash)
  d. Breed native tolerant ash
  e. Use accelerated breeding to breed native tolerant ash
  f. Cross breed native ash tree with non-native ash tree
  g. *cis*-Genesis (transferring genes between different species of ash)
  h. *trans*-Genesis (introducing a gene from another plant or animal into ash)
6. **Each option will require different lengths of time. Given this knowledge how, if at all, would your preferences change?**
  a. No action - let nature take its course (75 + years)
  b. Plant a different tree species e.g. oak (5 years)
  c. Plant different non-native species of ash (e.g. Manchurian tolerant ash) (5 years)
  d. Breed native tolerant ash (25 + years)
  e. Use accelerated breeding to breed native tolerant ash (15 + years)
  f. Cross breed native ash tree with non-native ash tree (20 + years)
  g. *cis*-Genesis (transferring genes between different species of ash) (5-10 years)
  h. *trans*-Genesis (introducing a gene from another plant or animal into ash) (5-10 years)
7. **Given that genetic modification can create a disease tolerant ash in 5-10 years time, while conventional breeding might take up to 30 years, would you object to the introduction of a genetically modified ash tree?**
  a. Planted in natural woodlands
  b. Planted in forestry plantations
8. **What is your attitude to genetically modified food/crops?**
  a. Support GM food/crops
  b. Anti GM food/crops
  c. Not sure/haven't thought about it
9. **Should the public have a say in which tree modification solution is adopted?**
  a. Yes, our views should be taken into account
  b. No, we should leave it to experts

## Appendix 2 Supplementary data

Questionnaire on tree-breeding solutions. Image 2
