# Supplementary material for: Developing publicly acceptable tree health policy: public perceptions of tree-breeding solutions to ash dieback among interested publics in the UK
Source: For Policy Econ. 2017 Jul;80:167–77. doi: 10.1016/j.forpol.2017.03.002 (PMC5473346; doi:10.1016/j.forpol.2017.03.002)
Supplement: Supplementary file 1 — Questionnaire on tree-breeding solutions. [file mmc1.docx]

## Supplementary material

**Figure 1. Responses to the question “*please rank your* *first and second most important source of information on countryside issues”*, % of respondents***

**multiple choice*

**Table 1. Respondents response to a question set measuring knowledge of tree identification and awareness of tree diseases effecting *F. excelsior* according to the age group and occupation**

|  | Correct identification of ash tree photo | Heard about ash dieback | Heard about *Chalara fraxinea* | Heard about emerald ash borer |
| --- | --- | --- | --- | --- |
| **Respondent’s age** | | | | |
| <21 | 47.6% | 68.2% | 4.5% | 27.3% |
| 21-30 | 67.4% | 82.6% | 25.0% | 20.7% |
| 31-40 | 48.1% | 75.9% | 13.9% | 21.5% |
| 41-50 | 53.8% | 85.4% | 14.6% | 21.0% |
| 51-60 | 69.0% | 93.6% | 18.4% | 19.5% |
| 61-70 | 67.9% | 95.1% | 15.7% | 16.2% |
| 71-80 | 71.4% | 94.7% | 19.8% | 28.2% |
| >81 | 64.3% | 86.2% | 17.2% | 24.1% |
| **χ^2^** | 51.577*** | 57.384*** | 9.280 | 10.059 |
| **Work sector** |  |  |  |  |
| Transport, retail or wholesale | 50.0% | 82.4% | 9.8% | 9.8% |
| Business, finance and insurance | 52.6% | 88.5% | 11.5% | 19.2% |
| Manufacturing, construction and agriculture and forestry | 64.5% | 91.1% | 24.8% | 27.4% |

**Table 2. Preferred solutions to ADB (before timescale) (number of respondents reported in brackets)**

| **Course of action** | **1^st^ choice** | **2^nd^ choice** | **3d choice** |  | **2^nd^ least preferred choice** | **Least preferred choice** |
| --- | --- | --- | --- | --- | --- | --- |
| No action (let nature take its course) | 3.7% (31) | 2.5% (21) | 8.3% (70) |  | 29.5% (250) | **56.1% (475)** |
| Plant a different tree species, e.g.oak | 9.3% (62) | 15.4% (103) | 27.4% (183) |  | 34.4% (230) | 13.3% (89) |
| Plant non-native species of ash | 7.3% (32) | 17.5% (77) | 24.0% (106) |  | 31.3% (138) | 20.0% (88) |
| Breed native tolerant ash | **68.8% (720**) | 22.3% (233) | 8.1% (85) |  | 0.6% (6) | 0.1% (1) |
| Accelerated breeding of native tolerant ash | 27.0% (237) | **55.5% (487)** | 14.7% (129) |  | 1.7% (15) | 1.0% (9) |
| Cross breed native ash with non-native ash | 6.3% (35) | 22.4% (125) | **56.5% (315)** |  | 8.6% (48) | 6.1% (34) |
| Cis-genetics | 3.9% (21) | 14.2% (77) | 36.0% (195) |  | 36.3% (197) | 9.6% (52) |
| Trans-genetics | 0.8% (5) | 2.2% (14) | 7.5% (48) |  | **55.7% (357)** | 33.9% (217) |

**Table 3. Chi-square test results between respondents’ characteristics, and their 3 most preferred and 2 least preferred solutions to deal with ADB (timescale considered)**

|  | **Potential options to deal with ADB** | | | | |
| --- | --- | --- | --- | --- | --- |
|  | 1^st^ most preferred option | 2^nd^ most preferred option | 3d most preferred option | Least preferred option | Second least preferred option |
|  | Breeding native tolerant ash | Accelerated breeding | Cross-breeding | No action – let nature take its course | Trans-genetics |
| **Gender** | | | | | |
| Male | 49.3% | 42.7% | 53.8% | 55.3% | 54.9% |
| Female | 47.3% | 41.7% | 47.9% | 55.9% | 53.5% |
| p-value | .059 | .428 | .150 | .238 | .306 |
| **Generation** | | | | | |
| Generation Y or Millennials | 43.3% | 44.3% | 32.7% | 60.4% | 33.3% |
| Generation X | 48.8% | 33.9% | 40.7% | 60.7% | 54.8% |
| Baby boomers | 49.3% | 45.0% | 55.1% | 52.2% | 58.2% |
| Pre-war generation | 49.2% | 41.5% | 61.4% | 48.0% | 57.0% |
| p-value | .000*** | .044 | .007 | .062 | .002** |
| **Level of Education** | | | | | |
| GCSE/A-levels or equivalent | 51.1% | 43.8% | 53.2% | 51.0% | 58.1% |
| Vocational training or similar | 46.7% | 41.9% | 52.6% | 40.9% | 72.1% |
| University degree or equivalent | 45.8% | 41.0% | 50.9% | 59.0% | 48.9% |
| p-value | .069 | .837 | .842 | .005** | .000*** |

**Figure 2a, b. Attitude to Genetically modified ash trees planted in natural woodlands (a; n=1139) and forestry plantations (b; n=1136), % of respondents**

**a)**

**Table 4. Chi-square test results on the relationship between attitude to GM ash trees planted in natural woodlands, and ranking of cis-genetics and trans-genetics options to treat Ash dieback**

|  | **Attitude to GM ash trees planted in natural woodlands** | | |
| --- | --- | --- | --- |
|  | Approve or strongly approve | Neither approve nor object | Object or strongly object |
| **Cis-genetics** | | | |
| 1 | 22.1%(51) | 13.7%(20) | 5.8%(13) |
| 2 | 19.0%(44) | 18.5%(27) | 8.4%(19) |
| 3 | 29.9%(69) | 28.8%(42) | 16.4%(37) |
| 7 | 22.1%(51) | 30.8%(45) | 58.4%(132) |
| 8 | 6.9%(16) | 8.2%(12) | 11.1%(25) |
| χ^2^ | 86.711*** | | |
|  | Approve or strongly approve | Neither approve nor object | Object or strongly object |
| **Trans-genetics** | | | |
| 1 | 3.2%(7) | 0.7%(1) | 0.4%(1) |
| 2 | 13.9%(30) | 5.8%(8) | 2.1%(6) |
| 3 | 12.0%(26) | 8.0%(11) | 3.2%(9) |
| 7 | 30.6%(66) | 34.8%(48) | 25.8%(73) |
| 8 | 40.3%(87) | 50.7%(70) | 68.6%(194) |
| χ^2^ | 67.238*** | | |

*Note: *** significant at 1% level*

**Table 5. Chi-square test results on the relationship between attitude to GM ash trees planted in forestry plantations, and ranking of cis-genetics and trans-genetics options to treat Ash dieback**

|  | **Attitude to GM ash trees planted in forestry plantations** | | |
| --- | --- | --- | --- |
|  | Approve or strongly approve | Neither approve nor object | Object or strongly object |
| **Cis-genetics** | | | |
| 1 | 19.0%(68) | 7.5%(10) | 5.5%(6) |
| 2 | 18.2%(65) | 15.7%(21) | 3.7%(4) |
| 3 | 28.9%(103) | 25.4% (34) | 11.9%(13) |
| 7 | 25.8%(92) | 41.8%(56) | 68.8%(75) |
| 8 | 8.1%(29) | 9.7%(13) | 10.1%(11) |
| χ^2^ | 80.722*** | | |
|  | Approve or strongly approve | Neither approve nor object | Object or strongly object |
| **Trans-genetics** | | | |
| 1 | 2.3%(8) | 0.0%(0) | 0.7%(1) |
| 2 | 10.5%(37) | 1.5%(2) | 3.4%(5) |
| 3 | 9.9%(35) | 5.2%(7) | 2.8%(4) |
| 7 | 30.5%(108) | 32.8%(44) | 24.1%(35) |
| 8 | 46.9%(166) | 60.4%(81) | 69.0%(100) |
| χ^2^ | 39.078*** | | |

*Note: *** significant at 1% level*
